# Metal Electrodes for Filtering the Localized Fundamental Mode of a Ridge Optical Waveguide on a Thin Lithium Niobate Nanofilm

**DOI:** 10.3390/nano13202755

**Published:** 2023-10-13

**Authors:** Mikhail Parfenov, Petr Agruzov, Aleksandr Tronev, Igor Ilichev, Anna Usikova, Yurii Zadiranov, Aleksandr Shamrai

**Affiliations:** Ioffe Institute, Politekhnicheskaya 26, 194021 St. Petersburg, Russia

**Keywords:** integrated photonics, thin-film lithium niobate, optical waveguide, modulators, mode filtering

## Abstract

An approach for filtering the fundamental mode in an integrated optical modulator with multimode waveguides based on etched thin lithium niobate nanofilms is presented. It is shown that metal electrodes can be used as a modal filter to suppress high-order modes in wide multimode ridge waveguides and, consequently, to provide their quasi-single-mode regime of operation. The influence of the gap between the electrodes and its displacement relative to the waveguide symmetry axis is analyzed for various configurations of waveguides. The conditions for quasi-single-mode light propagation with suppression of high-order modes of more than 90 dB/cm are found. The influence of fabrication errors on the efficiency of modal filtering is discussed. Efficient electro-optical modulation with an equivalent voltage-length product of 4 V∙cm has been experimentally demonstrated on integrated optical phase modulator samples fabricated using conventional contact photolithography. The proposed topological solution can be further used for the fast and cheap fabrication of TFLN modulators by conventional contact photolithography. The proposed modal filtering can also be used in other waveguide topologies and in more complex waveguide devices.

## 1. Introduction

Lithium niobate (LN, LiNbO_3_) has been one of the most attractive materials in integrated photonics for several decades [1,2,3] due to its large transparency window and its electro-optical and non-linear optical properties enabling fast light control and switching. Widespread commercially available broadband LN modulators are based on optical waveguides fabricated using titanium diffusion or proton exchange [1,2], with relatively weak light localization and large transverse mode sizes of about 10 μm.

With the commercialization of lithium-niobate-on-insulator (LNOI) wafers [4,5] optical waveguides can be fabricated on thin (≈300–700 nm) nanofilms of lithium niobate (TFLN—thin-film lithium niobate), which increases the transverse localization of light to the submicron level due to high refractive index contrast [6,7,8,9]. This new nanomaterial has greatly advanced integrated LN photonics. Significantly higher light localization than in conventional diffused LN waveguides provides high integration density, higher performance, lower cost, and new functionality of integrated optical devices that are compelling advantages in applications ranging from optical communications and sensors to microwave and quantum photonics.

At present, a wide variety of integrated optical devices based on the TFLN platform have been developed, such as electro-optical modulators [10,11], tunable interleavers [12,13], ring cavities [14,15], tunable Bragg filter gratings [16,17], wavelength converters and parametric amplifiers [18,19], lasers [20,21], nonclassical generators of light and entangled photon pairs [22,23], and many others. Most of them demonstrate sizes and performance unattainable for conventional integrated optical devices based on bulk lithium niobate substrates.

The efficient operation of integrated optical devices requires single-mode propagation of light through waveguides, which can be achieved if the transverse size of the waveguide is small enough. While the localization of light in TFLN waveguides is determined by the height of the etched ridge in the vertical dimension, the localization in the horizontal dimension is limited by the width of the waveguide and, hence, by the resolution of the lithography.

Most of the demonstrated TFLN integrated optical devices have been realized by employing electron-beam lithography, which provides the necessary resolution and is very effective for device prototyping but is a serial patterning technique that requires a prohibitively long write time, which is a major challenge for scaling. As was recently demonstrated, deep ultraviolet (DUV) lithography solves this problem and can be used for the fabrication of photonic integrated schemes (PICs) on 4- and 6-inch LNOI wafers [24]. However, DUV lithography has a high cost of ownership due to expensive resistive materials and laser maintenance, so its use is only reasonable for mass production. At present, the total volume of production of TFLN-integrated optical devices is quite small; therefore, the possibility of using traditional contact photolithography, which is used to manufacture integrated optical devices on substrates of bulk crystalline lithium niobate, looks very attractive. Some steps in this direction have been made earlier [25,26].

The low resolution of standard contact photolithography makes it impossible to fabricate single-mode waveguides on LNOI substrates that support only the fundamental mode. Waveguides which are easy to fabricate using standard contact photolithography have rather large widths (>1 μm) and, as a result, are multimode [27]. However, fundamental mode filtering can be implemented, given that it must be more localized in the waveguide than high-order modes.

Different waveguide structures can be potentially used for selective influence on optical modes and, in particular, for suppression of high-order modes in multimode waveguides. The typical approach for the induction of losses in optical dielectric waveguides is placing conducting layers near waveguides [28]. In general, the presence of conducting layers in waveguide devices can be effectively used for controlling light propagation. For example, conducting layers can be used for polarization filtering in optical waveguides or waveguide attenuators [28,29]. Moreover, conducting layers can provide isolation between waveguiding layers [30].

The goal of this work was to investigate the possibility of using metal electrodes of an electro-optical integrated optical phase modulator fabricated on an LNOI substrate using traditional contact photolithography for filtering the fundamental mode and the suppression of high-order modes.

## 2. Influence of Electrodes on Light Propagating in an Optical Waveguide

### 2.1. Considered Configuration

**Figure 1:**
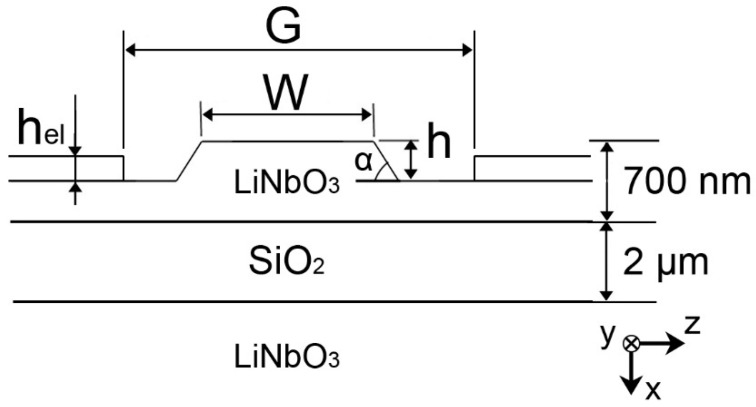
Waveguide phase shifter cross-section.

The basic element of an optical modulator is a phase shifter, which has the configuration of a straight waveguide surrounded by electrodes (Figure 1). In the case of an LNOI, a ridge waveguide is usually formed by etching to a depth h of a thin-film layer of lithium niobate (TFLN) lying on an intermediate silicon dioxide (SiO_2_) bonding layer (LNOI wafers from NanoLN). A typical cross-section profile has a sidewall angle α, which depends on the etching technology and mask material.

For the theoretical analysis, the following ridge cross-section was chosen: the thickness of the TFLN was 700 nm, the thickness of the silicon dioxide (SiO_2_) bonding layer was 2 µm, the waveguide ridge height h was varied in the range of 200 ÷ 400 nm, and the sidewall angle α = 25° corresponds to the chromium (Cr) mask and the inductively coupled plasma (ICP) dry etching process [31]. TFLN was considered in an x-cut orientation, which is usually used for the fabrication of electro-optical modulators. Aurum with a complex refractive index (1.5785–15.658 i [32]) was chosen as the electrode material. The analysis was performed at the wavelength of λ = 1550 nm for the TE modes which can experience the highest electro-optic coefficient in the considered crystal orientation. The influence of the ridge width W and the interelectrode gap on the mode composition of the waveguide was analyzed.

The mode composition of the waveguide was analyzed numerically using the finite-element method in the COMSOL Multiphysics software package. To reduce computational complexity, we neglected the interaction and transformation of two orthogonal polarization modes (TE and TM); therefore, a birefringent crystal was considered as an isotropic material with an extraordinary refractive index n_e_ = 2.14 for TE modes and the ordinary refractive index n_o_ = 2.21 for TM modes [2].

### 2.2. Waveguide Mode Filtering

**Figure 2:**
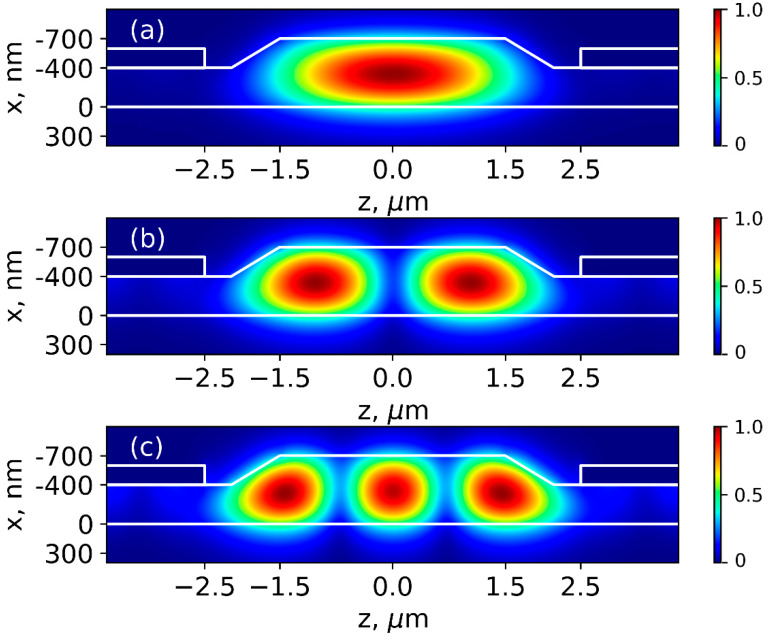
Spatial electric field distributions of TE waveguide modes (at W = 3 μm, h = 300 nm, G = 5 μm) (**a**)—fundamental mode (TE00 mode), (**b**,**c**)—high-order modes (TE10 and TE20, respectively).

Three ridge widths W were considered: 1.5, 2, and 3 μm, which can be easily produced using contact photolithography, but they certainly support the propagation of high-order modes [27]. The field of modes is not confined to the ridge and penetrates the surrounding TFLN layer (Figure 2). High-order modes penetrate the TFLN layer to a greater extent and, therefore, experience additional optical losses due to their interaction with metal electrodes. In this regard, using electrodes was proposed for filtering the fundamental mode which is more strongly localized within the ridge waveguide. The interelectrode gap (G) is a parameter that determines the filtration efficiency. In turn, the height of electrodes h_el_ (for values of h_el_ larger than 100 nm required for low ohmic losses) does not affect the filtration efficiency since it significantly exceeds the depth of light penetration into the metal. Therefore, the interelectrode gap size (G) was varied and the height of electrodes h_el_ = 200 nm was fixed.

**Figure 3:**
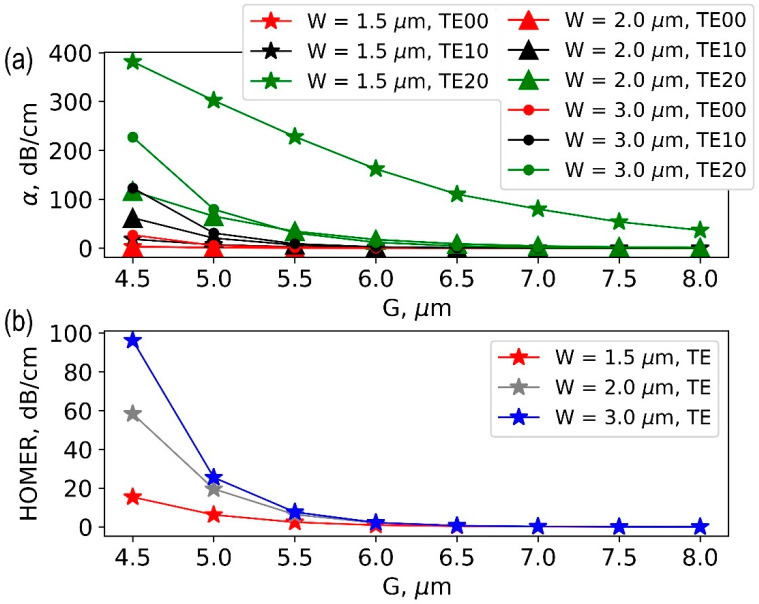
(**a**) Dependence of mode propagation losses on the interelectrode gap (G) for ridge waveguides with height h = 300 nm and different ridge widths (W); (**b**) HOMER as function of the interelectrode gap (G) at different ridge widths (W).

The results of the numerical calculation of the absorption coefficient for the fundamental mode and the nearest high-order mode at a fixed ridge height h = 300 nm and different widths W are shown in Figure 3. At the large interelectrode gap G = 8 um, the fundamental and high-order modes have low propagation losses. Note that the analysis considered ideal waveguides without defects and scattering losses, so propagation loss is entirely due to absorption at the interface with the metal. As the interelectrode gap decreases, propagation losses increase. The higher the order of modes, the higher the growth of losses.

The quantitative criterion for modal filtering is the high-order mode extinction ratio (HOMER), which can be expressed in decibels as the difference between the absorption coefficients of the nearest high-order mode ($\alpha_{\mathrm{TE}10}$) and the fundamental mode ($\alpha_{\mathrm{TE}00}$):

(1) $$\mathrm{HOMER}=\alpha_{\mathrm{TE}10}-\alpha_{\mathrm{TE}00}\left[\mathrm{dB}/\mathrm{cm}\right]$$

The calculated HOMER depending on the interelectrode gap G is shown in Figure 3b.

Highly efficient mode filtering with HOMER above 90 dB can be achieved at G = 4.5 μm for a ridge waveguide with h = 300 nm and W = 3 μm. The interelectrode gap in the range G = 4 ÷ 5 μm is a typical value for broadband modulators [10,11] and provides good velocity matching with acceptable impedance matching for high frequency modulation.

It was surprising that the waveguide with a wider ridge width W = 3 μm is more suitable for mode filtering and gives a higher HOMER. We attributed this to the behavior of the intensity cross-section of the fundamental mode, which is smaller for W = 3 µm than for 2 µm and 1.5 µm; in addition, the penetration of high-order modes into the surrounding TFLN layer is significant.

An increase in HOMER is accompanied by a simultaneous increase in losses of the fundamental mode. In general, losses in TFLN waveguides mainly depend on propagation losses due to scattering caused by the roughness of the waveguide edges and on coupling loss between optical waveguides and optical fibers because of modal size mismatch. Scattering leads to additional losses which depend on the overlap between the field of the waveguide mode and the edges of the waveguide, so they are higher for high-order modes. Therefore, scattering increases the differential mode losses caused by the metal electrodes and enhances the mode-filtering effect. However, scattering losses also lead to an increase in propagation losses of the fundamental waveguide mode, both due to direct scattering and due to additional losses at the metal electrodes, which has a negative effect on the proposed method of waveguide mode filtering with metal electrodes. Quantifying the losses caused by scattering will require additional detailed studies of the structure and morphology of the ridge waveguide. Just note that additional losses added to the fundamental mode by electrodes do not exceed typical losses obtained for TFLN waveguides nowadays [16].

**Figure 4:**
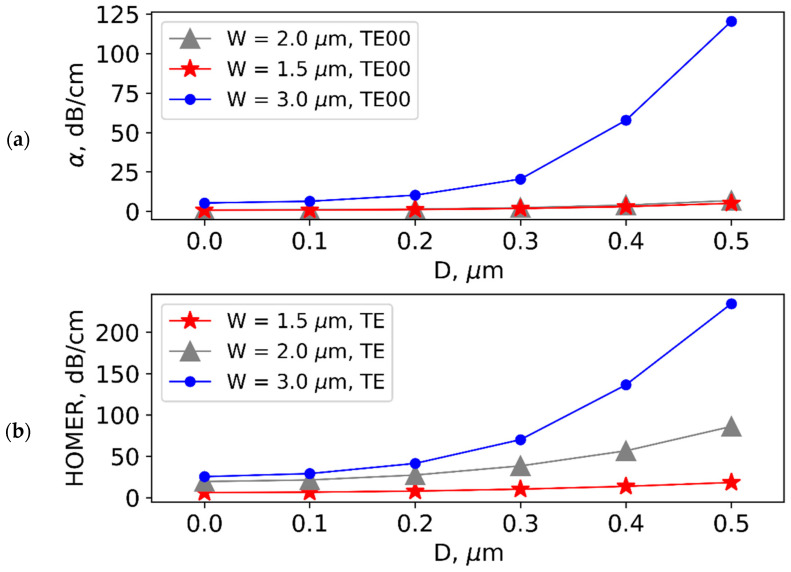
Dependence of (**a**) propagation losses of fundamental modes and (**b**) HOMER on displacement (D) between interelectrode gap center and optical waveguide symmetry axis for different waveguide ridge widths (W) at h = 300 nm.

Considering the limited resolution of photolithography, it should also be necessary to consider that a displacement between the interelectrode gap center and the waveguide symmetry axis can occur. Taking this factor into account, we numerically estimated the change in propagation losses of fundamental modes and in HOMER due to the possible displacement. The results obtained are presented in Figure 4. The results of the performed simulation show that displacement errors will even increase the efficiency of modal filtering.

### 2.3. Influence on Half-Wave Voltage

Reducing the interelectrode gap (G) not only provides mode filtering but should also increase the modulation efficiency. The product of the half-wave voltage (Vπ) of the modulator and the length (L) of its electrodes (VπL) was also analyzed. The VπL parameter can be calculated as

(2) $$V\pi L=\frac{\;\lambda }{n_{e}^{3}r_{33}\frac{\iint E_{\mathrm{el}}E_{\mathrm{opt}}^{2}\mathrm{dS}}{\iint E_{\mathrm{opt}}^{2}\mathrm{dS}}}\;$$

where r_33_ = 30 pm/V is the electro-optic coefficient in the waveguide propagating along the *y*-axis at the x-cut LNOI substrate and E_el_ and E_opt_ are cross-sections of the spatial distribution of the electric and optical fields, respectively. The values of relative permittivity ε_xLN_ = 35, ε_zLN_ = 95, ε_SiO2_ = 3.9 [33] were used in the calculations.

**Figure 5:**
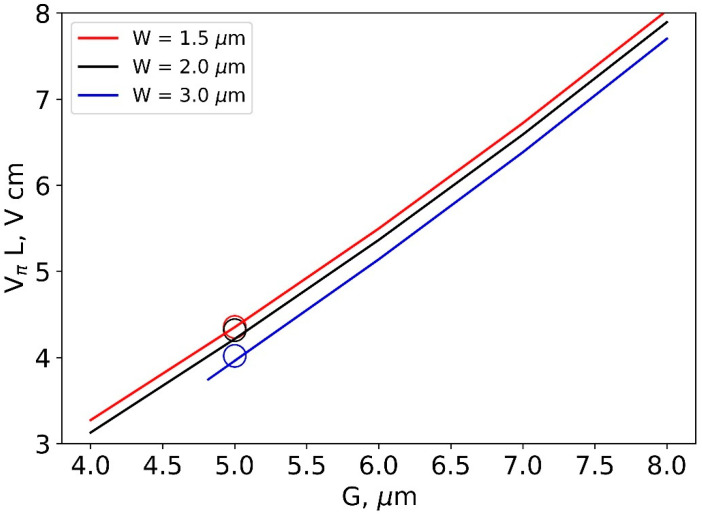
Dependence of half-wave voltage on the interelectrode gap size for different waveguide ridge widths (W) at its height h = 300 nm. The results of the experiment are marked with circles.

As expected, the half-wave voltage decreases as the interelectrode gap decreases. A slight decrease in VπL for the increased ridge width W = 3 μm provides additional merit besides the high-order mode filtering (Figure 5).

## 3. Experimental Investigations

### 3.1. Experimental Samples

To prove the proposed concept, samples of phase shifters (integrated optical modulators) were fabricated using contact photolithography. The configuration of the samples copied the configuration considered in the numerical simulation. Commercially available LNOI substrates from NanoLN with a TFLN layer thickness of 700 nm were used. Ridge waveguides were produced using the inductively coupled plasma (ICP) setup (“STE ICP 200e” from SemiTEq, Saint Petersburg, Russian) through a 150 nm thick chromium (Cr) mask. The etching rate was 25 nm/min. The width of the ridges was repeated in the same way as in the numerical model (1.5, 2, and 3 μm). The etching depth h = 300 nm was chosen based on the results of the numerical analysis. Low-frequency planar 200 nm thick gold electrodes with a chromium adhesive sublayer (10 nm) were deposited with DC magnetron sputtering on each side of the ridge waveguides. The interelectrode gap G was 5 µm. The length of the electrodes L was 12 mm.

**Figure 6:**
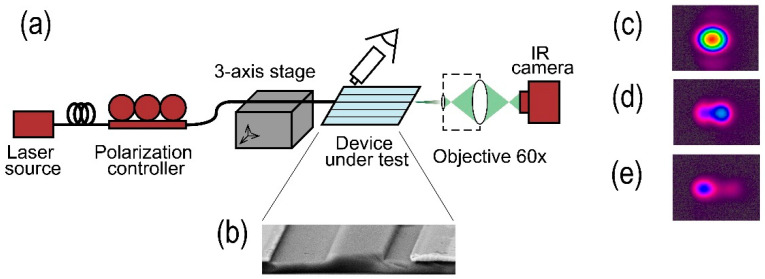
(**a**) Experimental setup for observation of modes on the waveguide output and examples of captured intensity distributions (for the waveguide at W = 3 um, h = 300 nm; (**b**) electron microscopy image of a fabricated sample (waveguide of the width W = 1.5 μm surrounded by electrodes with interelectrode gap G = 5 μm); (**c**) image of the captured fundamental TE mode; (**d**,**e**) images with excited high-order TE modes).

The electron microscopy image of one of the samples is shown in Figure 6. The rough sidewalls of the ridge indicate imperfect etching conditions which require further elaboration. Therefore, the optical losses of the samples were quite high—around 30 dB on the sample 20 mm in length—including input/output coupling losses with lensed optical fibers. Note that sidewall roughness could add a contribution to the mode filtering which has not been taken into account in the theoretical analysis.

### 3.2. Observation of High-Order Mode Suppression

To observe the waveguide modes, the setup shown in Figure 6 was used. A distributed feedback laser diode (DFB LD) with an output power of 20 mW with single-mode fiber output was used as a light source at the wavelength λ = 1550 nm. The TE polarization of light at the input of the waveguide was set by a fiber-optic polarization controller. A lensed optical fiber (with spot diameter of 2 µm and working distance of 12 µm) was used to launch light into ridge waveguides. The near-field image of the intensity distribution at the output of the waveguides was formed in an IR CCD camera (SPIRICON SP-503U-1550) with the help of a 60× micro-objective. The rather low resolution of the system did not allow a quantitative characterization of HOMER but gave a qualitative assessment of the presence or absence of high-order modes.

The presence of high-order modes manifests itself in a change in the intensity distribution when the tip of the lensed optical fiber is displaced relative to the input of the ridge waveguide, which is detected with IR CCD (see Figure 6). This change is the result of a change in the efficiency of high-order mode excitation, which transforms the interference pattern of the waveguide modes at the output. An evident presence of high-order modes was observed for experimental samples with waveguides without electrodes. Only a decrease in intensity without a change in the spatial distribution was observed for waveguides surrounded by electrodes with G = 5 μm, which indicates an efficient suppression of high-order modes.

### 3.3. Half-Wave Voltage Measurement

**Figure 7:**
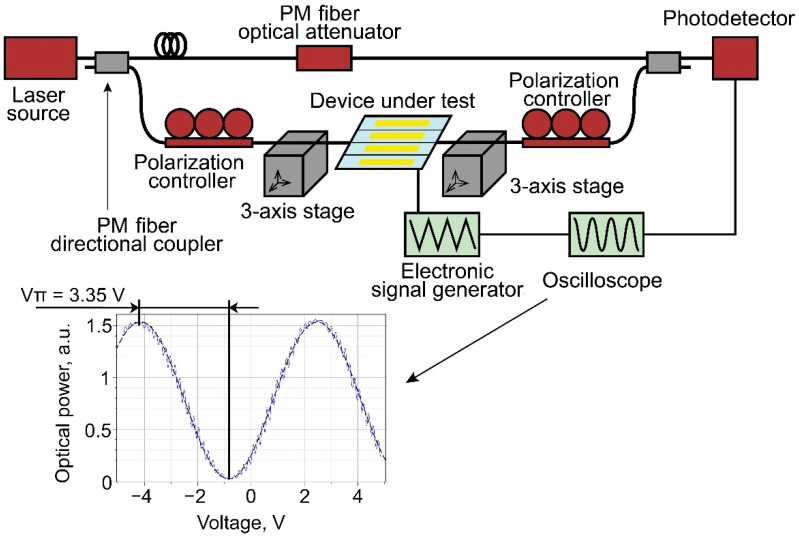
Setup with PM fiber-optical interferometer used for half-wave voltage measurements and example of V_π_ measurement (for a modulator with electrode length L = 12 mm).

The efficiency of the electro-optical modulation was then tested. The phase modulator was inserted into one of the arms of a Mach–Zehnder interferometer based on polarization-maintaining (PM) fibers (Figure 7).

Since the lensed fibers used for light coupling at the input and at the output of the fabricated samples were not polarization-maintaining, two fiber optic polarization controllers at the input and output of the modulator were used. The first controller was used to set linear TE polarization of light at the input of the waveguide; the second one oriented linear polarization at the output in accordance with the slow axis of the PM fiber. Triangular signal at a frequency of 1 kHz was applied to the modulator electrodes. In the second arm of the interferometer, a polarization-maintaining optical attenuator was used to adjust the interferogram contrast to a maximum. The interferogram had a regular sinusoidal shape, which is additional evidence for the effective suppression of high-order modes.

It is difficult to conduct a rigorous statistical analysis because only a few samples were produced due to the complexity of the fabrication process. However, experimental tests of the samples showed reproducible results.

The results of the measurements of half-wave voltages are in good agreement with the predictions of the numerical simulation. A slight decrease in the half-wave voltage was also observed with an increase in the width (W) (Figure 5).

## 4. Discussion

We used the simplest topology of an integrated optical modulator (straight waveguide phase shifter) for a demonstration of the concept of waveguide mode filtering and high-order modes suppression. The conditions for achieving a quasi-single-mode regime of operation in waveguides with a large cross-section and the possibility of its implementation were analyzed and experimentally demonstrated.

To focus the attention on the losses induced by narrow electrodes, scattering losses were not considered in the analysis. However, the presence of scattering losses will increase the propagation losses in addition to the losses induced by metal electrodes. It is well known that the scattering losses are caused by the roughness of waveguide edges [34], thus the value of scattering losses depends on the overlap between the optical mode field and the waveguide edges, which is larger for high-order modes. As a consequence, scattering losses will increase the differential modal losses caused by metal electrodes and will enhance the modal filtering effect.

Note that the sidewall angle had a fixed value in the analysis. The technology of waveguide fabrication requires the etching of thin films of lithium niobate. In turn, the etching of lithium niobate provides waveguide sidewalls with sufficient slope. It leads to narrowing the gap between electrode edges and waveguide edges. An increase in sidewall angle can be interpreted as a decrease in the effective width of the waveguide ridge and vice versa, which was analyzed in this manuscript.

It should be mentioned that, for practical implementation, possible errors in waveguide topology should be taken into account, in particular, deviations of waveguide ridge width, interelectrode gap, and displacement between the waveguide symmetry axis and the center of the interelectrode gap. The efficiency of mode filtering depends on these parameters which cannot be precisely controlled in the case of contact photolithography with a rather low resolution. However, the calculated dependences show that efficient suppression of high-order modes is possible, even assuming possible errors.

The waveguide topology analyzed in this paper had a narrow interelectrode gap and a wider waveguide ridge width than in standard topologies used in integrated optical TFLN modulators. The choice of fabrication methods was typical for standard integrated optical devices based on TFLN. Aurum, which was considered in this manuscript as the electrode material, is being used for the fabrication of electrodes not only on thin lithium niobate films but also on bulk lithium niobate substrates. Moreover, aurum electrodes are being used in commercially available electro-optic modulators on lithium niobate substrates [35]. Thus, the considered waveguide device has no potential technological problems, and there is no need for additional testing under temperature and environmental conditions. As expected, the stability parameters of the device with the proposed topology will be the same as those for standard integrated optical TFLN modulators.

It should be pointed out that the investigation was devoted to the induction of losses in waveguide modes and a planar electrode configuration which is not suitable for high-frequency modulation was considered. However, the proposed topology is compatible with high-frequency modulator topologies. The interelectrode gap value G = 5 μm which was chosen for the experimental samples is typical for coplanar electrodes optimized for high-frequency optical modulators based on TFLN. The height of high-frequency electrodes is also usually higher than that in fabricated samples (≈1–2 μm in contrast to h_el_ = 200 nm used in this work), and, as mentioned earlier, the considered effect is not dependent on electrodes’ heights since it significantly exceeds the depth of light penetration into the metal.

In general, the presented method of mode filtering was considered for the simplest waveguide topology but it has great potential for further investigations of its applicability in various more complicated structures. The influence of metal electrodes on mode filtering can be considered for other integrated optical devices like ring resonators, power splitters, filters, etc.

Note that the optimal configuration which has been found for straight waveguides should be optimized for other waveguide devices with a more complicated topology. For example, wide and consequently multimode waveguides are often being used in ring resonators for nonlinear optical applications [36]. Modes of curved multimode waveguides have shifted optical modal fields and, consequently, slightly other configuration of electrodes is necessary in this case.

It should also be mentioned that methods for selective influence on the modal compositions of waveguides can be used not only for the fabrication of quasi-single-mode waveguides using wide waveguides but also for the control of other parameters of waveguide devices. For example, it can potentially be used for tuning a splitting ratio in power splitters due to the different light spatial field distributions of symmetric and antisymmetric modes in directional couplers [37]. In y-branches, where power splitting is performed not through optical coupling but through wavefront division, the presence of metal layers deposited near the branching region can potentially lead to the induction or suppression of the antisymmetric mode and, in turn, to power splitting ratio change [38]. Moreover, the influence of mode filtering can be investigated not only for etched TFLN waveguides but also for hybrid waveguides on TFLN and for waveguides based on alternative material platforms, such as silicon, silicon nitride, or A3B5 waveguides.

## 5. Conclusions

The results obtained show that filtering of a strongly localized fundamental mode and effective suppression of weakly localized high-order modes of ridge TFLN waveguides is possible with closely spaced metal electrodes.

The dependence of losses induced in the optical modes of an etched ridge TFLN waveguide on the size of the interelectrode gap was calculated and analyzed for different waveguide ridge widths. The influence of fabrication defects on optical losses and high-order mode suppression including displacement errors in the proposed topology was estimated. Additional aspects concerning the choice of materials, electrode configuration, influence of fabrication errors and scattering losses, ways for optimization, and scientific perspectives were considered and discussed.

Conditions for the quasi-single-mode operation regime of an optical waveguide were determined. It was shown that the proposed topology can provide suppression of high-order modes of more than 90 dB/cm. Experimental samples of integrated optical phase modulators with the proposed topology were fabricated and tested. The observation of intensity spatial distribution on the waveguide output confirmed the results of the performed numerical investigation and proved the possibility of mode filtering by means of metal electrodes. The performance of the fabricated experimental samples was tested in a fiber-optic interferometer. The measured voltage-length product of the modulators was ≈4 V∙cm, which corresponds to the typical values known from the literature on phase modulators based on TFLN [31].

Thus, the possibility of fabricating TFLN modulators with multimode waveguides initially and subsequent mode filtering is demonstrated. Moreover, it was shown that, in contrast to the conventional approach which requires the fabrication of narrow waveguides using high-resolution electron-beam lithography or deep-ultraviolet photolithography, the approach based on mode filtering in wide and initially multimode waveguides can be considered as an alternative simple and cost-effective fabrication technique. Its potential for efficient mode filtering and high-order mode suppression was shown to be not only valuable for lower technological requirements but also sufficiently tolerant of fabrication errors.

Note that the proposed method is potentially applicable in other waveguide topologies and in more complex waveguide devices.

The considered topological solutions can be further used for the fast and cheap fabrication of TFLN modulators using conventional contact photolithography. The proposed mode filtering can be further used in other waveguide topologies and in more complex waveguide devices.

## Data Availability

The data presented in this study are available upon request from the corresponding author.
